# Transcript Profiling Analysis and ncRNAs’ Identification of Male-Sterile Systems of *Brassica campestris* Reveal New Insights Into the Mechanism Underlying Anther and Pollen Development

**DOI:** 10.3389/fpls.2022.806865

**Published:** 2022-02-08

**Authors:** Dong Zhou, Caizhi Chen, Zongmin Jin, Jingwen Chen, Sue Lin, Tao Lyu, Dandan Liu, Xinpeng Xiong, Jiashu Cao, Li Huang

**Affiliations:** ^1^Laboratory of Cell & Molecular Biology, Institute of Vegetable Science, Zhejiang University, Hangzhou, China; ^2^Hainan Institute of Zhejiang University, Sanya, China; ^3^Institute of Life Sciences, Wenzhou University, Wenzhou, China; ^4^College of Bioengineering, Jingchu University of Technology, Jingmen, China

**Keywords:** *Brassica campestris*, pollen development, long non-coding RNA, miRNA, endogenous target mimic, miRNA precursor

## Abstract

Male-sterile mutants are useful materials to study the anther and pollen development. Here, whole transcriptome sequencing was performed for inflorescences in three sterile lines of Chinese cabbage (*Brassica campestris* L. ssp. *chinensis* Makino, syn. *B. rapa* ssp. *chinensis*), the genic male-sterile line (A line), the *Polima* cytoplasmic male-sterile (CMS) line (P line), and the *Ogura* CMS line (O line) along with their maintainer line (B line). In total, 7,136 differentially expressed genes (DEGs), 361 differentially expressed long non-coding RNAs (lncRNAs) (DELs), 56 differentially expressed microRNAs (miRNAs) (DEMs) were selected out. Specific regulatory networks related to anther cell differentiation, meiosis cytokinesis, pollen wall formation, and tapetum development were constructed based on the abortion characteristics of male-sterile lines. Candidate genes and lncRNAs related to cell differentiation were identified in sporocyteless P line, sixteen of which were common to the DEGs in *Arabidopsis spl*/*nzz* mutant. Genes and lncRNAs concerning cell plate formation were selected in A line that is defected in meiosis cytokinesis. Also, the orthologs of pollen wall formation and tapetum development genes in *Arabidopsis* showed distinct expression patterns in the three different sterile lines. Among 361 DELs, 35 were predicted to interact with miRNAs, including 28 targets, 47 endogenous target mimics, and five precursors for miRNAs. Two lncRNAs were further proved to be functional precursors for bra-miR156 and bra-miR5718, respectively. Overexpression of *bra-miR5718HG* in *B. campestris* slowed down the growth of pollen tubes, caused shorter pollen tubes, and ultimately affected the seed set. Our study provides new insights into molecular regulation especially the ncRNA interaction during pollen development in *Brassica* crops.

## Introduction

Anther development starts from an archesporial cell that produces sporogenous cells that further differentiate into the endothecium, middle layer, tapetum, and pollen mother cells (PMCs) (Murmu et al., 2010). In *Arabidopsis*, *Sporocyteless*/*Nozzle* (*SPL/NZZ*) is the key regulator of anther cell differentiation, whose mutation blocks the formation of sporocytes (Yang et al., 1999). The basic leucine-zipper transcription factors *TGACG motif-binding protein 9* (*TGA9*) and *TGA10* work downstream of *SPL/NZZ* and interact with floral CC-type glutaredoxins ROXY1 and ROXY2 (Murmu et al., 2010). Ath-miR156 is downregulated in *spl/nzz* and its overexpression in *spl8* mutant leads to similar phenotypes of *spl/nzz* mutant (Xing et al., 2010). Although several key regulators have been found, molecular networks controlling anther cell specification remain largely unknown.

Pollen development can be divided into two major phases: the developmental phase and the functional phase (Hafidh and Honys, 2021). The latter refers to the interaction between pollen and stigma in which pollen grains rehydrate and germinate with pollen tubes to accomplish double fertilization (Johnson et al., 2019). The developmental stage of pollen is initiated by the meiosis of diploid PMCs. Tetrads formed by four haploid microspores are generated after simultaneous-type cytokinesis in dicotyledon, which means cell plates form between four haploid nuclei simultaneously (De Storme and Geelen, 2013). The position of the meiotic cell plate is determined by radial microtubule arrays, a phragmoplast-like structure consisting of actin filaments and microtubules. In *Arabidopsis*, there is a classical mitogen-activated protein kinase (MAPK) cascade signaling pathway, *Kinesin-like protein NPK1-activating kinase 1/2* (*AtNACK1*/*2*)-*Arabidopsis NPK1-related protein kinase* (*ANP1*/2/*3*)-*MAPK kinase* (*MAPKK6*)-*MAPK4*, regulating mitotic cell plate expansion *via* affecting the phosphorylation of microtubule crosslinker protein MAP65s (De Storme and Geelen, 2013). Notably, *AtNACK2*, *MAPKK6*, and *MAPK4* also function in male-specific meiotic cytokinesis while no evidence showed that ANP1 and ANP3 work in male meiosis as yet (Yang et al., 2003; Zeng et al., 2011). Thus, the MAPK cascade is still incomplete for male meiosis. During somatic cytokinesis, the Exocyst complex regulates fusion of clathrin-coated vesicles in the midzone and compromised initial cell plate assembly was observed in the Exocyst complex subunit EXO70A1 mutants (Fendrych et al., 2010). In contrast to mitotic cytokinesis, little is known about the regulatory network underlying plant male meiotic cytokinesis.

The second meiotic division is usually accompanied by the formation of a callose wall (Shen et al., 2019). *Callose synthase 5* (*Cals5*) is the key gene responsible for the synthesis of callose surrounding tetrads (Dong et al., 2005). *Arabidopsis Ruptured pollen grain 1* (*RPG1*), a sugar transporter encoding gene, shares a redundant function with *RPG2* in regulating the expression of *Cals5* (Sun et al., 2013). The callose wall is degraded by callose secreted by the tapetum to release free microspores. Tapetum is the last structure surrounding the developing PMCs and microspores, supplying essential substances and enzymes required for microsporogenesis and pollen maturation. A well-defined genetic pathway, *Dysfunctional tapetum 1* (*DYT1*)-*Defective in tapetal development and function 1* (*TDF1*)-*Aborted microspores* (*AMS*)-*MYB domain protein 80* (*MYB80*)-*MS1* has been identified for tapetal development (Parish and Li, 2010). *Vanguard 1* (*VGD1*) and *Glyoxal oxidase 1* (*GLOX1*) are direct targets of *MYB80* and may be involved in the formation of the pollen coat (Phan et al., 2011). Three cysteine proteases [Carbohydrate epimerase 1 (CEP1), RD19A, and RD19C] play irreplaceable roles in tapetal PCD and their maturation is regulated by β vacuolar processing enzyme (βVPE) (Cheng et al., 2020). After the degradation of the callose wall, the synthesis of the pollen wall starts. The pollen wall includes an inner intine mainly composed of pectin, cellulose, and hemicellulose, and an outer exine mainly composed of sporopollenin (Jiang et al., 2013). Pectin and cellulose are synthesized from sucrose by enzymes, such as cell wall invertases (cwINVs) and fructokinases (FRKs), in microspores (Shi et al., 2015). Sporopollenin is synthesized in the tapetum and secreted to the surface of the microspore to form exine. Although the exact structure of sporopollenin remains unknown, several genes like *Arabidopsis male sterility 2* (*MS2*), *acyl-CoA synthetase 5* (*ACOS5*), and *cytochrome p450 family 703 subfamilies A polypeptide 2* (*CYP703A2*) have been revealed to involve in its synthesis (Shi et al., 2015).

In recent years, a vital role of non-coding RNAs (ncRNAs) including microRNAs (miRNAs) and long ncRNAs (lncRNAs) have been revealed in reproductive processes. For example, overexpression of bra-miR158 reduced the pollen viability and pollen germination ratio in *B. campestris* (Ma et al., 2017). Reduced expression of *Long-day-specific male-fertility-associated RNA* (*LDMAR*) triggered premature PCD of tapetum and then resulted in male sterility in rice under long-day conditions (Ding et al., 2012). In addition, interactions between ncRNAs have also been discovered: lncRNAs can act as miRNA decoys that are also known as endogenous target mimics (eTMs). Excessive expression of *osa-eTM160* impaired the repression of osa-miR160 on its targets, resulting in less seed set in rice (Wang et al., 2017). Our previous study also predicted 15 lncRNAs as the potential eTMs for 13 miRNAs during pollen development and fertilization in *B. campestris* and demonstrated that identified two functional eTMs for miR160 in pollen development (Huang et al., 2018). LncRNAs can also act as miRNA targets and precursors. In sweet orange (*Citrus sinensis*), three lncRNAs, *csi-eTM166*, *XLOC_009399* (a precursor of csi-miR166c), and *XLOC_016898* (a target of csi-miR166c) together with csi-miR166c could form an eTM166-miR166c-targeted lncRNA regulatory network, which possibly affected citrus fruit development (Ke et al., 2019). To uncover the interactions between different types of transcripts is undoubtedly important to interpret gene expression networks during anther and/or pollen development.

With anther and/or pollen abortion as the most common variable defect, male-sterile lines are suitable materials to study the molecular and cellular mechanisms underlying anther and pollen development. According to the mode of inheritance, male sterility can be divided into genic male-sterile (GMS) and cytoplasmic male-sterile (CMS). The former is controlled by nuclear genes and the latter is caused by mitochondrial genes together with nuclear genes. *Polima* (*Pol*) and *Ogura* (*Ogu*) CMS are widely used in the breeding of *Brassica* crops. In *Pol* CMS lines, sporogenous cells fail to differentiate into the endothecium, middle layer, and tapetum, and ultimately no pollen sac is formed (An et al., 2014). In *Ogu* type CMS lines, the obvious malformation is discovered during the late tetrad and uninucleate stage where tapetal cells are radially expanded and vacuolated, and the deposition of sporopollenin is delayed (Kang et al., 2014; Xing et al., 2018). As a typical GMS line, Chinese cabbage (*B. campestris* ssp. *chinensis* cv. Aijiaohuang) ‘*Bcajh97-01A*’ has been demonstrated to undergo aberrant cytokinesis during male meiosis with defective exine formation, and premature tapetal PCD (Huang et al., 2009; Shen et al., 2019). To systematically explore the molecular mechanisms underlying anther/pollen development in *Brassica* crops, we created the GMS line ‘*Bcajh97-01A*,’ a *Pol* CMS line ‘*Bcpol97-05A*,’ and an *Ogu* CMS line ‘*Bcogu97-06A*’ of Chinese cabbage (hereafter called A line, P line, and O line, respectively) that shared the same maintainer line ‘*Bcajh97-01B*’ (B line) by successive selection and back-crossing (Huang et al., 2008; Liang et al., 2019). In this study, whole transcriptome sequencing was performed to disclose the regulatory networks between mRNAs and ncRNAs, and interactions of different types of ncRNAs during anther and/or pollen development in *B. campestris*. Differentially expressed genes (DEGs) and ncRNAs associated with pollen development were identified in different sterile lines. We also constructed the interplay between lncRNAs and miRNAs, which will broaden our knowledge on the molecular mechanisms underlying male sterility and proceed with its utilization in breeding.

## Materials and Methods

### Plant Materials

The *Ogu* CMS line (*B. napus*) was transferred into ‘*Aijiaohuang*’ (*B. campestris*) through successive backcrossing four times. And then, the fertile plant (B line) in the ‘*Aijiaohuang*’ two-type line ‘*Bcajh97-01A/B*’ were backcrossed to *Ogu* CMS in *B. campestris* for four generations. The fertility and morphology characteristics like height and width of backcross progeny were observed over successive years. Finally, we established an O line that shared the common maintainer line (B line) with the A line.

The three sterile lines (A line, P line, and O line) and their common maintainer line (B line) in Chinese cabbage were cultivated in the experimental farm of Zhejiang University, Hangzhou, China at the same time. Inflorescences were harvested from plants at the full flowering stage with the removal of open flowers. Each sample of inflorescences was collected from more than 10 individual plants, frozen in liquid nitrogen immediately, and stored at −75°C for further use. Three replicates were performed.

### Whole Transcriptome Sequencing

Total RNA was extracted using TRIzol reagent (Invitrogen, Carlsbad, CA, United States) following the manufacturer’s instructions. Libraries for mRNA-Seq and lncRNA-Seq were generated using NEBNext^®^ Ultra*™* Directional RNA Library Prep Kit for Illumina^®^ (NEB, Ipswich, MA, United States) as the instruction manual described. Libraries for miRNA-Seq were generated using NEBNext^®^ Ultra*™* small RNA Sample Library Prep Kit for Illumina^®^ (NEB) following the manufacturer’s recommendations and index codes were added to attribute sequences to each sample. RNA-Seq was performed by the Biomarker Technologies Co., Ltd., Beijing, China^1^ on an Illumina Hi-Seq platform.

### Identification of ncRNAs and Differential Expression Analysis

The transcriptome was assembled using the StringTie package based on the reads mapped to the reference genome downloaded from the Brassica database, Institute of Vegetables and Flowers, Beijing, China (version 1.5, BRAD)^2^ and annotated using the gffcompare program. Coding-Non-Coding Index (CNCI), coding potential assessment tool (CPAT), coding potential calculator (CPC), and Pfam were used to predict lncRNAs. The secondary structure of lncRNAs was generated by the Mfold web server.^3^ Known miRNAs were identified by aligning the mapped reads with the mature miRNAs’ sequences in miRBase, University of Manchester, Manchester, United Kingdom (version 21).^4^ Reads were identified as known miRNAs when the alignment was identical. Novel miRNAs were predicted by a modified miRDeep2, Max Delbrück Center for Molecular Medicine, Berlin, Germany with plant-specific parameters (version 2.0.5). DESeq R package, Dana-Farber Cancer Institute, MA, United States (version 1.10.1) was used for screening DEGs, differentially expressed lncRNAs (DELs), DECs, and differentially expressed miRNAs (DEMs). The criteria were a |fold change (FC)| ≥ 2 and a false discovery rate (FDR) ≤ 0.05.

### Construction of lncRNA-mRNA, lncRNA-miRNA, and Transcription Factors Networks

Adjacent genes within 100 kb of lncRNAs were considered to be their *cis*-targets and *trans*-targets, which were predicted by LncTar software (Casero et al., 2015). Pearson bivariate correlation was adopted to estimate the expression relationships between ncRNAs and their targets. The correlation is significant at a confidence level (bilateral) ≤ 0.05 (*P* < 0.05) and extremely significant when *P*-value ≤ 0.01. Targets of DEMs were predicated by TargetFinder, Beijing Institute of Radiation Medicine, Beijing, China (version 1.6). LncRNAs as potential miRNA targets were selected by the psRNATarget website^5^ with the default parameter. LncRNAs as miRNA precursors were predicted by comparing lncRNAs with miRNA precursor sequences. LncRNAs as miRNA eTMs were predicted by a local Perl script (Ye et al., 2014). The transcription factor network was constructed by iGRN, a newly developed tool for transcriptional networks, and visualized using Cytoscape, Institute for Systems Biology, Washington, WA, United States (version 3.8) (Meng et al., 2021).

### *Nicotiana benthamiana* Transient Expression Assay

Fragments of *bra-miR156HG* and *bra-miR5718HG* were amplified and inserted into the pBI121 vector under two CaMV 35S promoters, respectively. Primers used for vector construction are listed in Supplementary Table 1. Plasmids were transformed into *Agrobacterium tumefaciens* strain GV3101 and then infiltrated into tobacco leaves when OD_600_ reached 1.0–1.2. The leaves were collected, frozen in liquid nitrogen immediately, and stored at −75°C for RNA extraction after 48 h of infiltration.

### Semi-Quantitative and Real-Time Quantitative RT-PCR

For transient expression assay, semi-quantitative reverse transcription (RT)-PCR of lncRNAs was performed with 2 × TSINGKE master mix (Tsingke Biotechnology Co., Ltd., Beijing, China) and *nbe-18S rRNA* was used as a reference. Expression of miRNAs was detected by real-time quantitative PCR (RT-qPCR) on a CFX96 Real-Time System (Bio-Rad, Hercules, CA, United States) using TB Green Premix Ex Taq II (TaKaRa, Dalian, China) and *nbe-5.8S rRNA* was used for normalization. For validation of RNA-Seq, RT-qPCR was performed using the same samples for RNA-Seq. The expressions of DEGs and DELs were normalized against *BraUBC10* and those of DEMs were normalized against *bra-5.8S rRNA*. The relative expression levels were calculated using the 2^–ΔΔCT^ method. The cDNAs used for the RT-qPCR analysis of miRNA were synthesized by a Mir-X™ miRNA First-Strand Synthesis Kit (TaKaRa). All primers are shown in Supplementary Table 1.

### Data Availability

Transcriptome data supporting the findings of this study have been deposited in National Center for Biotechnology Information Sequence Read Archive (SRA) under the accession number PRJNA753197. All other relevant data are available from the corresponding author on request.

## Results

### Transcripts Were Identified Genome-Wide in *Brassica campestris*

**FIGURE 1:**
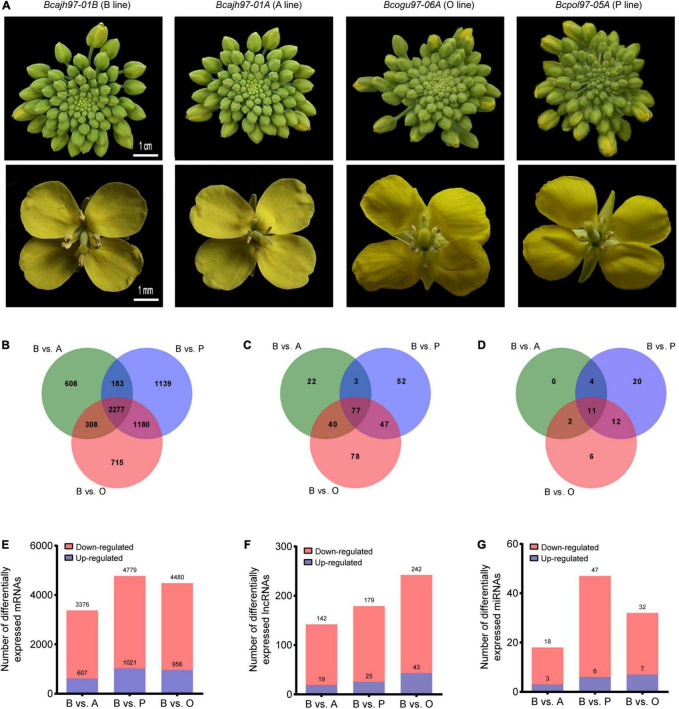
Genome-wide identification and characteristics of transcripts in *Brassica campestris*. **(A)** Photographs for inflorescences and flowers from the sterile and the fertile line. **(B,E)** Statistics of differentially expressed mRNAs in three sterile lines and the fertile line. **(C,F)** Statistics of differentially expressed long non-coding RNAs in three sterile lines and fertile line. **(D,G)** Statistics of differentially expressed microRNAs in three sterile lines and fertile line. B line, the maintainer line ‘*Bcajh97-01B*’; A line, the genic male-sterile line ‘*Bcajh97-01A*’; P line, *Polima* cytoplasmic male-sterile line ‘*Bcpol97-05A*’; O line, *Ogura* cytoplasmic male-sterile line ‘*Bcogu97-06A*’.

As the general morphology of inflorescences and flowers of the three sterile lines did not differ from those of the common fertile maintainer line except for the anthers, inflorescences without opened flowers of A line, P line, O line, and B line were taken for transcriptome sequencing to analyze the transcriptome during pollen and anther development (Figure 1A). For lncRNAs and mRNAs, a total of 141.89 GB of clean data were obtained for 12 samples, with an average of 10.20 GB of clean data per sample. For microRNA, a total of 253.31 M clean data were obtained, and clean reads of each sample no less than 14.40 M. A total of 41,133 mRNAs, 13,879 putative lncRNAs, and 342 miRNAs were identified (Supplementary Figures 1A–C). The identified lncRNAs were classified into lincRNAs, antisense lncRNAs, sense lncRNAs, and intronic lncRNAs, which were evenly distributed on different chromosomes (Supplementary Figures 1D,E). The expression difference of transcripts between each male-sterile line and the maintainer line was then compared respectively. Totally, 7,136 DEGs, 361 DELs, 56 DEMs were selected out (Figures 1B–D). A total of 2,277 DEGs, 77 DELs, and 11 DEMs were common to all sterile lines. Further, 608, 1,139, and 715 DEGs were specific to A line, P line, and O line, respectively (Figures 1B–D). As for DELs, 22, 52, and 78 DELs were specifically expressed in A line, P line, and O line, respectively. No line-specific DEMs were found in the A line, 20 DEMs were specifically expressed in the P line, and only six DEMs were specifically expressed in the O line. Compared with the B line, 3,376, 4,779, and 4,480 DEGs were identified in A line, P line, and O line, respectively, 79% of which on average were downregulated (Figures 1B,E). Among the three sterile lines, the largest number of DELs was discovered in O line (242), followed by P line (179), and the least was in A line (142) (Figures 1C,F). The number of miRNAs expressed in four lines was comparatively lower than other transcripts. There were 18, 47, and 32 differentially expressed microRNAs (DEMs) identified in A line, P line, and O line, respectively (Figures 1D,G). All the expression data of DEGs, DELs, and DEMs were concluded in Supplementary Data 1. Interestingly, like mRNAs, the proportion of downregulated non-coding transcripts in sterile lines including lncRNAs and miRNAs was significantly higher than that of upregulated transcripts (Figures 1E–G). Further, we carried out the RT-qPCR experiments of seven DEGs, six DELs, and three DEMs to validate the results of RNA-Seq. The results showed that the expression pattern of these candidate genes was consistent with the results of RNA-Seq, which suggest the transcriptome data were reliable (Supplementary Figure 2).

### Using the P Line We Found Candidate Transcripts Involved in Anther Cell Differentiation

**FIGURE 2:**
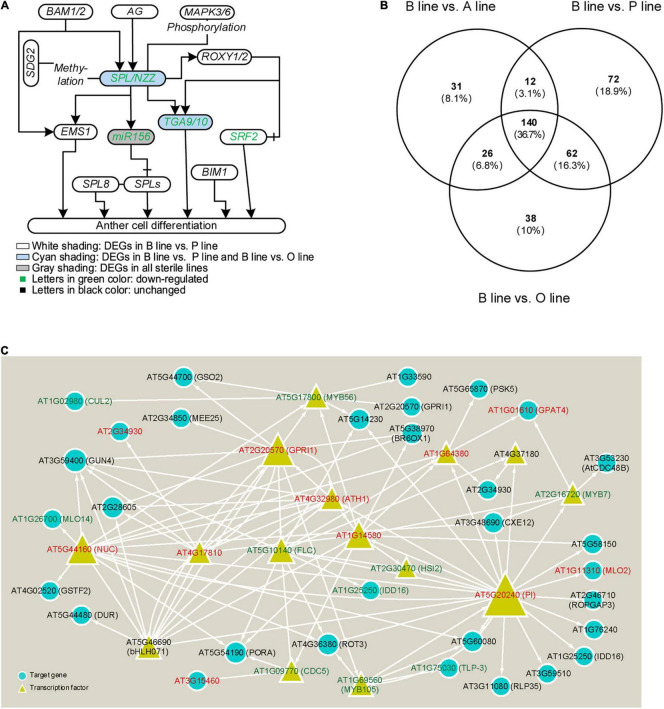
Analysis of genes in anther cell differentiation in three sterile lines of *Brassica campestris*. **(A)** Genic network of anther cell differentiation. **(B)** Venn diagram of differentially expressed genes (DEGs) number involved in cell differentiation in fertile line and sterile lines. **(C)** Regulatory network of transcription factors annotated to participate in cell differentiation predicted by integrated gene regulatory network (iGRN). Genes in red mean upregulated DEGs, while genes in green represent downregulated DEGs. B line, the fertile line ‘*Bcajh97-01B*’; A line, the sterile line ‘*Bcajh97-01A*’; P line, *Polima* (*Pol*) cytoplasmic male-sterile (CMS) line ‘*Bcpol97-05A*’; O line, *Ogura* (*Ogu*) CMS line ‘*Bcogu97-06A*’; BAM1/2, β-amylase 1/2; SDG2, SET domain protein 2; EMS1, excess microsporocytes 1; AG, agamous; SPL/NZZ, sporocyteless/nozzle; SPL8, SQUAMOSA promoter binding protein-like 8; MAPK3/6, mitogen-activated protein kinase 3/6; ROXY1/2, CC-type glutaredoxin 1/2; TGA9/10, TGACG (TGA) motif-binding protein 9/10; BIM1, brassinosteroid insensitive 1 (BRI1)-EMS-suppressor 1 (BES1)-interacting MYC-like protein 2; SRF2, STRUBBELIG-receptor family 2; CUL2, cullin 2; GUN4, genomes uncoupled 4; MLO2/14, mildew resistance locus O; NUC, nutcracker; GSTF2, Glutathione s-transferase phi 2; DUR, defective uge in root; bHLH071, basic helix-loop-helix protein 71; GSO2, gassho 2; MEE25, maternal effect embryo arrest 25; MYB7/56/105, MYB domain protein 7/56/105; GPRI1, GBF’s pro-rich region-interacting factor 1; FLC, flowering locus C; CDC5/48B, cell division cycle 5/48B; BR6OX1, brassinosteroid-6-oxidase 1; HSI2, high-level expression of sugar-inducible gene 2; IDD16, indeterminate(id)-domain 16; TLP-3, thaumatin-like protein 3; RLP35, receptor like protein 35; PSK5, phytosulfokine 5 precursor; GPAT4, glycerol-3-phosphate sn-2-acyltransferase 4; CXE12, carboxylesterase 12; PI, pistillata; ROPGAP3, ROP guanosine triphosphatase (GTPASE)-activating protein 3.

To excavate genes related to anther cell differentiation, firstly, the expression of genes that were experimentally confirmed to function in anther differentiation (reviewed by Walbot and Egger, 2016) was analyzed in the B line and the sterile lines. Our results showed that five out of them were DEGs in sterile lines compared with the fertile line (Figure 2A). Homolog genes of *Arabidopsis SPL/NZZ*, *Bra026359* (*NZZ-1*), and its putative downstream genes, *Bra018634* (*TGA9-1*), *Bra031622* (*TGA9-2*), *Bra009233* (*TGA10-1*), *Bra005914* (*TGA10-2*), and *Bra009231* (*STRUBBELIG-receptor family 2*, *SRF2*) were downregulated in P line, and so did miR156. While the expression of another homolog to *Arabidopsis SPL/NZZ*, *Bra019056* (*NZZ-2*) and four downstream genes except for *Bra009231* (*SRF2*) was also decreased in the O line and only miR156 was downregulated in the A line. Additionally, through target prediction and expression correlation analysis, five lncRNAs were found to target *SPL/NZZ* and its downstream genes (Supplementary Data 2). Two lncRNAs target *Bra009233* and one targets *Bra026359*, *Bra019056*, *Bra009233*, and *Bra005914*. *MSTRG.2229.1* was predicted to positively regulate *Bra026359* (*NZZ-1*) while *Bra019056* (*NZZ-2*) was negatively related with lncRNA *MSTRG.17700.1*. *MSTRG54890.1* was predicted to target *Bra009233* (*TGA10-1*).

Considering that molecular networks regulating anther cell differentiation are not well studied, it is reasonable that only several function-known DEGs related to it were identified in the three sterile lines. To further elucidate molecular networks in anther cell differentiation, all line-specific DEGs were filtered by gene ontology (GO) term “cell differentiation.” The number of DEGs annotated in P line (72) was approximately twice that of A line (31) and O line (38) (Figure 2B). For further analysis, only DEGs specifically expressed in the P line were included because a previous study described that sporogenous cells in *Pol* CMS anthers failed to differentiate, which was largely different from the phenotype of A line and O line (An et al., 2014). As *B. campestris* and *Arabidopsis* have a common ancestor, functional analysis of homologous genes in *Arabidopsis* can provide a reference to the function of genes in *B. campestris*. Integrated gene regulatory network (iGRN), a newly developed tool for transcription factors regulatory network prediction in *Arabidopsis* was used to investigate the relationship between homologs of the 72 P line-specific DEGs. Finally, 15 DEGs were screened out to form a regulatory network (Figure 2C). Twenty genes were found to interact with *Pistillata* (*PI*, *AT5G20240*) that controls the differentiation of petals and stamen, and 13 genes were found to interact with *Nutcracker* (*NUC*, *AT5G44160*) which affects flowering time *via* sugar metabolism (Bowman et al., 1989; Jeong et al., 2015). As the phenotype of the P line was similar to the *Arabidopsis spl/nzz* mutant, all P line-specific DEGs and the microarray data from anthers of *spl/nzz* were compared to discover more genes involved in anther cell differentiation (Wijeratne et al., 2007). Consequently, 16 common DEGs between *spl/nzz* and P line were identified and 13 of them showed the same expressional changes, among which six transcription factors (*FLC*, *bHLH071*, *ATH1*, *MYB56*, *MYB105*, and *MYB7*) were also identified with iGRN (Table 1). Five lncRNAs and three miRNAs were predicted to interact with seven DEGs (Supplementary Data 2). Notably, *Bra002042* (*MYB7*) were predicted to be the target of one lncRNA, *MSTRG.33978.1*, and two miRNAs, bra-miR159a and bra-miR319-3p (Supplementary Data 2).

**TABLE 1:** Common DEGs between *Polima* cytoplasmic male-sterile of *Brassica campestris* and the *Arabidopsis spl/nzz* mutant.

ID in BRAD	Expression in B line	Expression in P line	Regulated	Homologous genes in *Arabidopsis*		Gene annotation
				ID in TAIR	Gene name	
Bra021188	2.043	0.345	**Down**	AT3G16650	*Pleiotropic regulatory locus 2* (*PRL2*)	RNA processing and modification
Bra029913	56.944	6.537	**Down**	AT3G48690	*Carboxylesterase 12* (*CXE12*)	Serine hydrolases hydrolyzing 2,4-D-methyl
Bra002042	8.613	3.164	**Down**	AT2G16720	*MYB domain protein 7* (*MYB7*)	Cell differentiation, regulation of flavanol biosynthetic process
Bra009231	2.731	0.958	**Down**	AT5G06820	*Strubbelig-receptor family 2* (*SRF2*)	Protein phosphorylation
Bra006422	1.419	0.580	**Down**	AT5G17800	*MYB56*	Negative regulation of cell division
Bra008175	134.316	6.473	**Down**	AT1G75030	*Thaumatin-like protein 3* (*TLP-3*)	Defense response
Bra002513	1.757	0.045	**Down**	AT5G60080	-	Protein phosphorylation
Bra009055	2.000	0.198	**Down**	AT5G10140	*Flowering locus C* (*FLC*)	Floral transition repression and temperature compensation of the circadian clock
Bra003995	1.451	0.393	**Down**	AT1G69560	*MYB105*	Boundary specification, meristem initiation and maintenance, and organ patterning
Bra006978	1.560	0.197	**Down**	AT3G53230	*Cell division cycle 48B* (*CDC48B*)	Mitotic spindle disassembly
Bra039531	0.449	1.612	Up	AT5G44700	*Gassho2* (*GSO2*)	Regulation of cell division and cell fate specification
Bra003889	1.895	4.182	Up	AT1G72180	*C-terminally encoded peptide (CEP)* receptor 2 (*CEPR2*)	Receptor for CEP1 peptide
Bra028006	8.508	19.272	Up	AT1G33590	-	Response to Karrikin, signal transduction
Bra017533	0.699	2.011	**Up**	AT5G46690	*Basic helix-loop-helix protein 71* (*bHLH071*)	Interacting with FAMA to regulate stomatal differentiation
Bra026499	0.661	1.526	**Up**	AT5G23400	-	Signal transduction
Bra011403	2.550	5.431	**Up**	AT4G32980	*Arabidopsis thaliana homeobox 1* (*ATH1*)	Regulation of gibberellin biosynthetic genes

*The expression of genes was presented as fragments per kilobase of exon model per million mapped fragments. B line and P line refer to the fertile line ‘Bcajh97-01B’ and the Polima cytoplasmic male-sterile line ‘Bcpol97-05A’ of B. ampestris, respectively. Bold characters in the column “regulated” represent genes that showed the same expression change and non-bold ones represent genes that showed the opposite change in the Arabidopsis spl/nzz mutant and P line of B. ampestris. DEG, differentially expressed gene; TAIR, the Arabidopsis information resource; BRAD, Brassica database.*

### Utilizing A Line We Selected Potential Transcripts Related to Cell Plate Formation During Meiotic Cytokinesis

**FIGURE 3:**
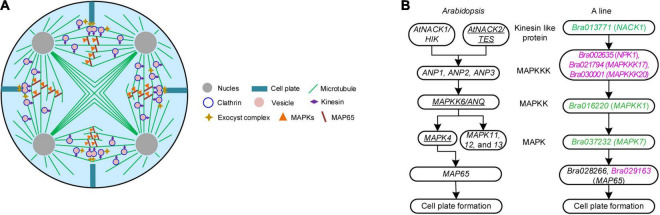
Pathways concerning meiosis cytokinesis in *Brassica campestris*. **(A)** Schematic diagram of simultaneous-type plant male cytokinesis. **(B)** Mitogen-activated protein kinase signaling cascade mediating *de novo* cell plate formation in *Arabidopsis* and the genic male-sterile line ‘*Bcajh97-01A*’ (A line) of *B. campestris*. The left was adapted from De Storme and Geelen (2013) and the right was a putative pathway hypothesized in this study. Underlined genes have been proved to function in male meiotic cytokinesis. Differentially expressed genes down and upregulated were colored in green and rose red, respectively. B line, the fertile line ‘*Bcajh97-01B*’; A line, the genic male-sterile line ‘*Bcajh97-01A*’; MAPKK, MAPK kinase; MAPKKK, MAPKK kinase; NACK1/2, Nicotiana protein kinase 1 (NPK1)-activating kinesin 1; HIK, hinkel; TES, tetraspore; ANP1/2/3, Arabidopsis NPK1-related protein kinase 1/2/3; ANQ, Arabidopsis Nicotiana kinase next to NPK1 (NQK1).

**FIGURE 4:**
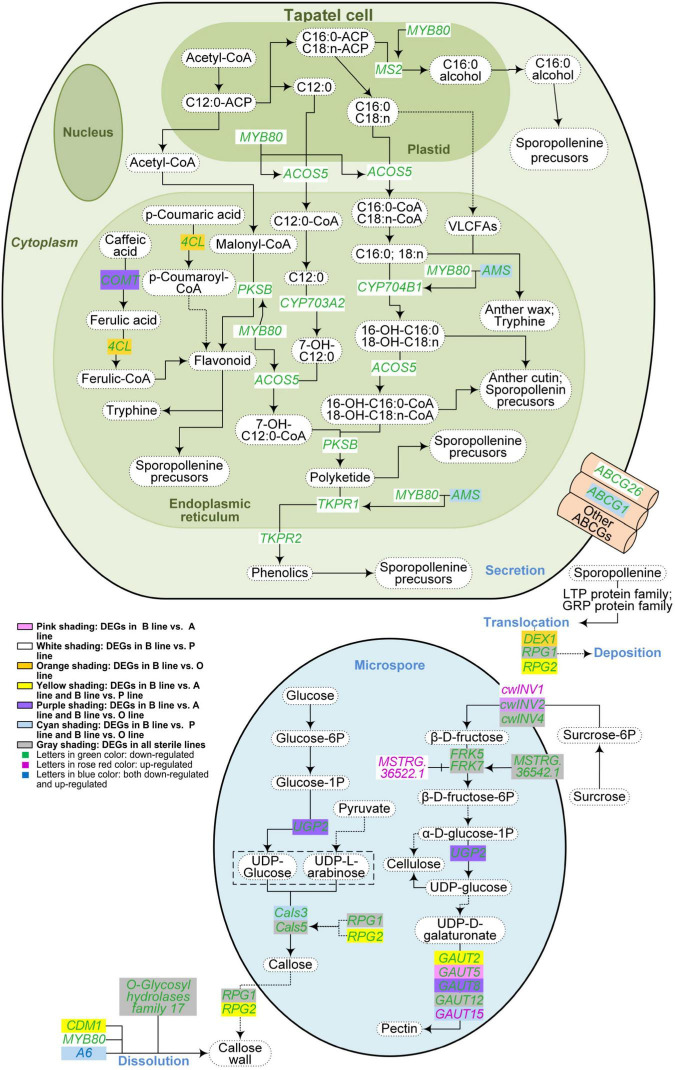
Metabolism pathways of pollen wall components in the three sterile lines of *Brassica campestris*. Differentially expressed genes associated with the metabolism of sporopollenin, cellulose, pectin, and callose were indicated in the figure. The solid arrows represent a direct relationship, and the dotted arrows indicate an indirect relationship. B line, the fertile line ‘*Bcajh97-01B*’; A line, the genic male-sterile line ‘*Bcajh97-01A*’; P line, the *Polima* cytoplasmic male-sterile line ‘*Bcpol97-05A*’; O line, the *Ogura* cytoplasmic male-sterile line ‘*Bcogu97-06A*’; MYB80, MYB domain protein 80; MS2, male sterility 2; ACOS5, acyl-CoA synthetase 5; COMT, caffeate *O*-methyltransferase; 4CL, 4-coumarate:CoA ligase; PKSB, polyketide synthase B; CYP703A2, cytochrome p450 family 703 subfamilies A polypeptide 2; CYP704B1, cytochrome p450 family 704 subfamily B polypeptide 1; AMS, aborted microspores; VLCFAs, very-long-chain fatty acids; TKPR1/2, tetraketide α-pyrone reductase 1/2; ABCG, Adenosine triphosphate (ATP)-binding cassette, subfamily G; UGP, uridine diphosphate (UDP)-glucose pyrophosphorylase; Cals, callose synthase; DEX1, defective in exine formation 1; RPG1/2, ruptured pollen grain 1/2; CDM1, callose defective microspore 1; cwINV, cell wall invertase; FRK, fructokinase; GAUT, galacturonosyl transferase.

Previous cytological observations showed that failure of tetrads formation in the A line was caused by the aberrant cytokinesis (Huang et al., 2009; Shen et al., 2019). To explore the network that participated in male meiotic cytokinesis, DEGs concerning it were selected *via* GO analysis, Swiss-Prot, and NR annotation. Finally, 13 DEGs, 11 of which were A line-specific and two were expressed in both A line and P line were identified (Table 2). Generally, dicotyledonous male meiocytes undergo simultaneous-type cytokinesis where cell plates determined by radial microtubule arrays form between all four haploid nuclei at the same time (Figure 3A). As one of the main components of radial microtubule arrays, actin plays an important role in cytokinesis (De Storme and Geelen, 2013). *Bra014865* (*ACT3*) and *Bra033236* (*ADF10*) annotated to encode an actin protein and an actin-depolymerizing factor 10, respectively, were downregulated in A line (Table 2). Clathrin light protein was reported to be the coating materials of vesicles secreted from the Golgi/Trans-Golgi network in somatic cytokinesis (Buschmann and Muller, 2019). In A line, two genes, *Bra031138* (*Clathrin light chain 1*, *CLC1*) and *Bra033472* (*CLC3*) were annotated to encode clathrin light protein (Table 2). During cytokinesis, a fusion of vesicles carrying substances required for cell plate synthesis is a crucial process, which is regulated by the Exocyst complex (De Storme and Geelen, 2013). Mutation of Exocyst complex subunit EXO70A1 is compromised the initial cell plate assembly (Fendrych et al., 2010). In A line, *Bra020294* was annotated as the Exocyst subunit exo70 and downregulated in A line (Table 2). Moreover, genes responsible for callose synthesis were downregulated in the A line as well (see below Figure 4). Three lncRNAs interacted with *Bra033472* (*CLC3*) and *Bra033236* (*ADF10*), two of which were targeted *Bra033472* (*CLC3*) (Supplementary Data 3).

**TABLE 2:** Specifically expressed genes involved in cytoplasmic cell plate formation in ‘*Bcajh97-01A*’ of *Brassica campestris*.

ID in BRAD	Expression in B line	Expression in A line	Regulated	Homologous genes in *Arabidopsis*		Swiss-Prot annotation	NR annotation
				ID in TAIR	Gene name		
Bra014865	50.911	25.592	Down	AT3G53750	Actin 3 (ACT3)	ACT3	ACT3
Bra033236	12.236	3.862	Down	AT1G01750	Actin-depolymerizing factor 10 (ADF10)	ADF10	A hypothetical ADF (CARUB_v10011760mg)
Bra020294	1.205	3.251	Up	AT5G59730	Exocyst subunit exo70 family protein H7 (EXO70H7)	–	EXO70H7
Bra031138	84.051	40.448	Down	AT2G20760	Clathrin light chain 1 (CLC1)	CLC1	A Hypothetical CLC protein (ARALDRAFT_900444)
Bra033472	2.534	6.372	Up	AT3G51890	CLC3	CLC3	CLC protein
Bra002635	0.141	0.672	Up	–	–	MAPKKK [Nicotiana protein kinase 1 (NPK1)]	Hypothetical MAPKKK A-like protein
Bra013771	0.638	0.255	Down	AT4G24170	–	Kinesin-like protein NPK1-activating kinase 1 (NACK1)	ATP binding microtubule motor family protein
Bra037232	2.729	1.305	Down	AT2G18170	Mitogen-activated protein kinase 7 (MAPK7)	MAPK7	P-loop containing nucleoside triphosphate hydrolases superfamily protein
Bra016220	2.731	1.439	Down	AT1G70430	–	MAPKK1	Kinase family protein
Bra021794	0.544	3.531	Up	AT2G32510	MAPKK kinase 17 (MAPKKK17)	MAPKKK3	MAPKKK17
Bra030001	0.187	0.927	Up	AT3G50310	MAPKKK20	MAPKKK20	MAPKKsK20
Bra034123	12.917	6.961	Down	AT3G10525	Siamese related 1 (SMR1)	Cyclin-dependent protein kinase inhibitor SMR1	Hypothetical protein (ARALDRAFT_478386)
Bra001367	12.750	6.011	Down	AT3G10525	SMR1	Cyclin-dependent protein kinase inhibitor SMR1	Unknown

*The expression of genes was presented as fragments per kilobase of exon model per million mapped fragments. B line, the fertile line ‘Bcajh97-01B’; A line, the genic male-sterile line ‘Bcajh97-01A’; TAIR, the Arabidopsis information resource; BRAD, Brassica database.*

In mitotic cytokinesis, the phosphorylation of MAP65 protein affects the expansion of phragmoplast, which is regulated by a classical MAPK cascade signaling pathway (De Storme and Geelen, 2013). To explore the MAPK pathway involved in male meiosis of *B. campestris*, DEGs specifically expressed in A line were screened out and a putative cascade signaling pathway was proposed according to the annotation of DEGs (Figure 3B). In this cascade signaling pathway, three MAPKKKs (NPK1, MAPKKK17, and MAPKKK20) worked downstream of *Bra013771*, which was annotated as the kinesin-like protein NACK1; then, three MAPKKKs targeted MAPKK1 that was encoded by *Bra016220*, and subsequently, MAPK7 was activated to phosphate the protein MAP65. Expression analysis showed that the expression of *Bra037232* encoding the protein MAPK7 was decreased (Supplementary Data 1). Eleven lncRNAs were presumed to target three MAPKKKs (Supplementary Data 3). Seven of them targeted *Bra021794* (*MAPKKK17*) while two targeted *Bra002635* (*NPK1*) and two targeted *Bra030001* (*MAPKKK20*), respectively (Supplementary Data 3).

### Transcripts Related to Pollen Wall Formation Showed Distinct Expression Patterns in Different Sterile Lines

Previous morphological observation showed that a rough and irregular surface rather than a reticulate exine structure existed on pollen grains in A line (Shen et al., 2019). Although the differentiation of sporogenous cells was arrested, a few anther sacs with withered pollen still reside in *Pol* CMS (An et al., 2014). In the *Ogu* CMS line, the exine of pollen was thinner than that in the fertile line (Lin et al., 2019). To explore the network regulating the pollen wall formation, the expression levels of genes involved in the metabolism of pollen wall components in the three sterile lines were analyzed. In this study, sporopollenin and callose synthesis and degradation pathways were concluded based on (Shi et al., 2015), and cellulose and pectin synthesis pathways were drawn according to the Kyoto Encyclopedia of Genes and Genomes (KEGG) analysis.

A total of 32 DEGs were selected out and 13 for sporopollenin synthesis and transportation, 9 for the synthesis and degradation of callose, and 11 of them were responsible for the biosynthesis of pectin and cellulose (Figure 4). Compared with the B line, genes involved in sporopollenin synthesis and transportation like homolog genes of *Arabidopsis MS2*, *ACOS5*, and *CYP703A2* were downregulated in the P line, while most of them showed no differential expression in the A line and O line. The expression level of *Bra037213*, orthologs of *Arabidopsis Cals5*, was decreased in all sterile lines. *Bra025595*, which is isogenous with *Arabidopsis RPG1*, was also reduced in all three sterile lines. However, *Bra022761*, orthologs of *Arabidopsis RPG2* that shares a redundant function with *RPG1*, was downregulated in A line and P line but not in O line. The expression of *Bra029746*, a syngenic gene of *Defective in exine formation 1* (*DEX1*) encoding a membrane calcium-binding protein that is required for the primexine matrix formation, was decreased only in the O line. *Bra000704* and *Bra003378* are orthologs of *Arabidopsis FRK5* and *FRK7* encoding fructokinases that phosphorylate fructose in cellulose and pectin synthesis, respectively, were downregulated in all sterile lines. Furthermore, ncRNAs interacting with DEGs in the synthesis and/or degradation metabolism pathways of sporopollenin, cellulose, pectin, and callose were predicted. Finally, 29 lncRNAs and one miRNA significantly correlated with corresponding DEGs were obtained (Supplementary Data 4). Fifteen DEGs were targeted by only one lncRNAs, three targeted by two lncRNAs, and two targeted by three lncRNAs. In contrast to the similar expression pattern between *MSTRG.16387.1* and *Bra013041* (*AMS*), *MSTRG.16374.1* showed the reverse expression trend with *Bra013041* (*AMS*).

### Tapetum Degradation-Related Genes Were Downregulated in Three Sterile Lines

**FIGURE 5:**
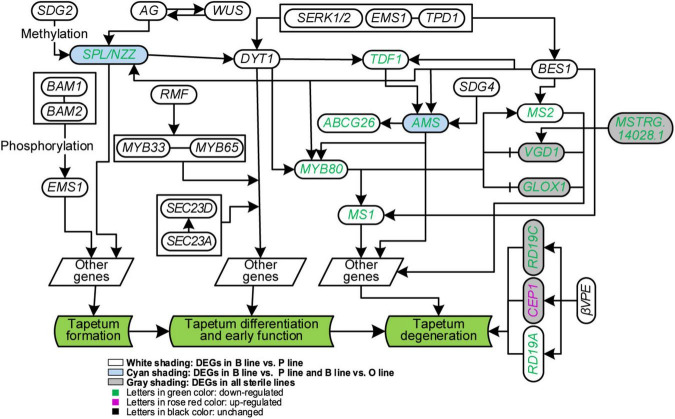
Analysis of genes in tapetum development of *Brassica campestris*. Genes in the same box were worked together to regulate downstream genes. B line, the fertile line ‘*Bcajh97-01B*’; P line, the *Polima* cytoplasmic male-sterile line ‘*Bcpol97-05A*’; O line, the *Ogura* cytoplasmic male-sterile line ‘*Bcogu97-06A*.’ SDG2/4, SET domain protein 2/4; BAM1/2, β-amylase 1/2; AG, agamous; WUS, wuschel; SPL/NZZ, sporocyteless/nozzle; RMF, reduced male fertility; MYB33/65, MYB domain protein 33/65; DYT1, dysfunctional tapetum 1; SERK1/2, somatic embryogenesis receptor-like kinase 1/2; EMS1, excess microsporocytes 1; TPD1, tapetum determinant 1; TDF1, defective in tapetal development and function 1; ABCG26, Adenosine triphosphate (ATP)-binding cassette subfamily G 26; AMS, Aborted microspores; MYB80, MYB domain protein 80; MS1/2, male sterility 1/2; VGD1, Vanguard 1; GLOX1, Glyoxal oxidase 1; CEP1, cysteine endopeptidase 1; βVPE, β vacuolar processing enzyme.

It has been reported that tapetum cells in A line exhibited premature PCD after meiosis (Shen et al., 2019), however, in *Ogu* CMS, tapetum cells became vacuolized and elongated radially at the uninucleate stage with delayed degradation (Kang et al., 2014; Xing et al., 2018). To unearth the underlying regulatory network of tapetum development, the expression of genes related to it in the three sterile lines was analyzed. Thirty protein-coding genes were included based on (Parish and Li, 2010). Among these genes, 12 of them were DEGs (Figure 5). Further analysis of gene expression changes showed that 11 of them were downregulated in the P line, half in the O line, and only four in the A line. Compared with the B line, all homogenous genes to the *DYT1-TDF1-AMS-MYB80-MS1* pathway except *Bra013519* (*DYT1*) were downregulated in the P line. Expression of genes correlated with tapetum formation and differentiation was not discrepant in the O line and A line, but the expression of genes interrelated with tapetum degradation declined in the two sterile lines. All copies of three homogenous genes of *Arabidopsis VGD1*, *GLOX1*, and *RD19C* relevant to tapetum degradation were downregulated, but *Bra000615*, a homolog to *CEP1*, was upregulated in the O line (Figure 5).

ncRNAs that interact with protein-coding genes in the network were selected out *via* target prediction and expression correlation analysis. Finally, 21 lncRNAs and one miRNA targeted 14 DEGs were obtained (Supplementary Data 5). The expression of *Bra025337* (*TDF1*) was negatively related to a newly defined miRNA, unconservative_A03_9803 (*P* = 0.039). Three lncRNAs, *MSTRG.33891.1*, *MSTRG.33874.2*, and *MSTRG.33884.1* were found to target the meristem control gene *Bra039894* (*WUS*).

### Extensive Interactions Between DELs and miRNAs Were Found During Anther and Pollen Development

LncRNAs and miRNAs have been shown to play critical roles in regulating gene expression *via* complex interaction networks (Han et al., 2021). To further investigate their functions in anther/pollen development, the interaction between DELs and miRNAs in the three sterile lines was explored. As a result, 35 DELs were predicted to interact with miRNAs, with most of which were identified in P line (23), followed by O line (21), and the least in A line (11) (Table 3). Twenty-eight DELs were putative targets of 43 miRNAs. Notably, six common DELs targeted by miRNAs were downregulated in the three sterile lines. Although 47 lncRNAs were predicted to work as eTMs for miRNA, only *MSTRG.30876.5* was DEL in the P line and O line, and no DEL functioned as eTM was found in the A line (Table 3 and Supplementary Data 6). Another six DELs were identified as host genes of nine miRNAs according to the similarity of sequences. Two lncRNAs, *MSTRG.50087.1* and *MSTRG.44699.1* (renamed as *bra-miR156HG* and *bra-miR5718HG*) were predicted to work as host genes for bra-miR156 and bra-miR5718, respectively (Supplementary Figure 3 and Supplementary Data 6).

**TABLE 3:** Summary of differentially expressed lncRNAs acting as miRNAs’ targets, precursors, and eTMs in *Brassica campestris*.

miRNA	Different roles of DELs								
	eTM			Target			Host gene		
	A line	P line	O line	A line	P line	O line	A line	P line	O line
Bra-miR156c	–	–	–	–	–	–	–	*MSTRG.50087.1*	–
Bra-miR156f	–	–	–	–	–	–	–	*MSTRG.50087.1*	–
Bra-miR158-5p	–	–	–	–	*MSTRG.20734.1*	*MSTRG.20734.1; MSTRG.20734.2*	–	–	–
Bra-miR162-5p	–	***MSTRG.*** ***30876.5***	*MSTRG.* *30876.5*	–	–	–	–	–	–
Bra-miR403-5p	–	–	–	***MSTRG.13936.3***	–	–	–	–	–
Bra-miR408-3p	–	–	–	*MSTRG.39657.1*	*MSTRG.39657.1*	–	–	–	–
Bra-miR5712	–	–	–	*MSTRG.6099.1*	*MSTRG.6099.1*	*MSTRG.6099.1*	–	–	–
Bra-miR5719	–	–	–	–	***MSTRG.55412.1***	***MSTRG.55412.1***	–	–	–
Bra-miR9408-3p	–	–	–	***MSTRG.12676.1***	–	–	–	–	–
Bra-miR9558-3p	–	–	–	–	–	***MSTRG.7520.4***	–	–	–
Bra-miR9562-5p	–	–	–	–	–	***MSTRG.7012.1***	–	–	–
Unconservative_A01_124	–	–	–		–	–	–	*MSTRG.50087.1*	–
Unconservative_A01_2822	–	–	–	***MSTRG.12676.1***	–	–	–	–	–
Unconservative_A01_337	–	–	–	*MSTRG.6099.1*	*MSTRG.6099.1*	*MSTRG.6099.1*	–	–	–
Unconservative_A01_3874	–	–	–	–	–	–	–	*MSTRG.10680.1; MSTRG.26529.1*	*MSTRG.10680.1*
Unconservative_A02_5263	–	–	–	–	*MSTRG.26116.1*	–	–	*MSTRG.21862.1*	–
Unconservative_A02_5624	–	–	–	–	–	***MSTRG.31712.2***	–	–	–
Unconservative_A02_9408	–	–	–	*MSTRG.47571.3*	***MSTRG.22869.1***	–	–	–	–
Unconservative_A02_9409	–	–	–	*MSTRG.47571.3*	***MSTRG.22869.1***	–	–	–	–
Unconservative_A03_10008	–	–	–	–	*MSTRG.20239.1*	–	–	–	–
Unconservative_A03_10228	–	–	–	*MSTRG.38026.1*	*MSTRG.38026.1*	*MSTRG.38026.1*	–	–	–
Unconservative_A03_10294	–	–	–	–	–	*MSTRG.17213.1*	–	–	–
Unconservative_A03_12816	–	–	–	*MSTRG.6099.1*	*MSTRG.6099.1*	*MSTRG.6099.1*	–	–	–
Unconservative_A03_13897	–	–	–	–	–	–	–	*MSTRG.50087.1*	–
Unconservative_A04_16774	–	–	–	–	***MSTRG.22869.1***	–	–	–	–
Unconservative_A04_16775	–	–	–	–	***MSTRG.22869.1***	–	–	–	–
Unconservative_A04_16989	–	–	–	–	–	–	–	–	–
Unconservative_A05_19537	–	–	–	***MSTRG.13936.3***	–	–	–	–	–
Unconservative_A05_21515	–	–	–	*MSTRG.16423.1*	*MSTRG.16423.1*	*MSTRG.16423.1*	–	–	–
Unconservative_A06_22943	–	–	–	–	***MSTRG.22869.1***	–	–	–	–
Unconservative_A06_23503	–	–	–	–	*MSTRG.26116.1*	–	–	–	–
Unconservative_A06_25032	–	–	–	–	***MSTRG.34516.1***	***MSTRG.34516.1*** *; MSTRG.25360.2*	–	–	–
Unconservative_A06_25702	–	–	–	–	***MSTRG.22869.1***	–	–	–	–
Unconservative_A07_27582	–	–	–	–	*MSTRG.41319.2*	*MSTRG.41319.2*	–	–	–
Unconservative_A07_28347	–	–	–	–	*MSTRG.56775.1*	–	***MSTRG.*** ***25551.2***	*MSTRG.25551.3; MSTRG.25550.1*	*MSTRG.25550.1; MSTRG.25551.1; MSTRG.25551.3*
Unconservative_A07_29448	–	–	–	–	***MSTRG.22869.1***	–	–	–	–
Unconservative_A07_29750	–	–	–	–	***MSTRG.8026.1***	–	–	–	–
Unconservative_A07_29886	–	–	–	–	***MSTRG.8026.1***	–	–	–	–
Unconservative_A08_30911	–	–	–	***MSTRG.27755.1***	–	–	–	–	–
Unconservative_A08_33222	–	–	–	–	–	*MSTRG.25360.2*	–	–	–
Unconservative_A09_33893	–	–	–	–	***MSTRG.22869.1***	–	–	–	–
Unconservative_A09_36589	–	–	–	–	***MSTRG.22869.1***	–	–	–	–
Unconservative_A09_36656	–	–	–	–	–	–	–	*MSTRG.50087.1*	–
Unconservative_A09_40282	–	–	–	–	***MSTRG.22869.1***	–	–	–	–
Unconservative_A09_40283	–	–	–	–	***MSTRG.22869.1***	–	–	–	–
Unconservative_A10_40369	–	–	–	*MSTRG.51803.1*	*MSTRG.51803.1*	*MSTRG.51803.1*	–	–	–
Unconservative_A10_41812	–	–	–	–	***MSTRG.22869.1***	–	–	–	–
Unconservative_A10_41813	–	–	–	–	***MSTRG.22869.1***	–	–	–	–
Unconservative_A10_42634	–	–	–	–	***MSTRG.22869.1***	–	–	–	–
Unconservative_A10_42699	–	–	–	–	*MSTRG.56864.1*	*MSTRG.56864.1*	–	–	–
Unconservative_scaffold 036722_49811	–	–	–	–	*MSTRG.21147.1*	–	–	–	–

*Long non-coding RNAs (lncRNAs) in bold font were upregulated and lncRNAs non-bolded were downregulated in sterile lines. eTMs, endogenous target mimics; A line, the genic male-sterile line ‘Bcajh97-01A’; P line, the Polima cytoplasmic male-sterile line ‘Bcpol97-05A’; O line, the Ogura cytoplasmic male-sterile line ‘Bcogu97-06A’.*

**FIGURE 6:**
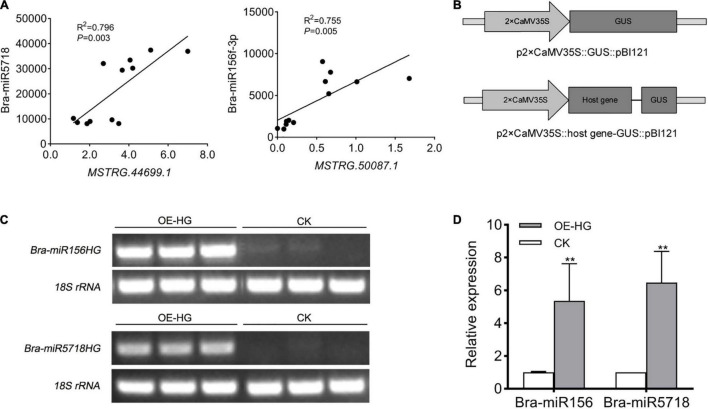
LncRNAs, *bra-miR156HG* and *bra-miR5718HG*, worked as host genes for miR156 and miR5718 in *Brassica campestris*. **(A)** Expression correlation between *bra-miR5718HG* and *bra-miR156HG* and the corresponding miRNAs. **(B)** Structure of expression vectors used for transient agroinfiltration assay. **(C)** Semi reverse transcriptase PCR verifying the overexpression of lncRNAs in tobacco leaves. The lane of OE-HG represents samples containing corresponding lncRNA overexpression vectors. **(D)** Real-time quantitative PCR revealing the up-regulation of miR156 and miR5718. Data were presented as mean ± SD and the *t*-test was used for significance analysis. **P < 0.01. Triplicate experiments were conducted. LncRNAs, long non-coding RNAs; miRNAs, microRNAs.

miR156 is a conserved miRNA in plants, which is widely known to participate in diverse processes of plant growth and development, especially vegetative phase change in *Arabidopsis* (He et al., 2018). miR5718 has been reported to respond to heat stress in *B. campestris* (Yu et al., 2012). In the inflorescences of *B. campestris*, the expression pattern of these two lncRNAs was positively correlated with that of *bra-miR156* (*P* = 0.005) and *bra-miR5718* (*P* = 0.003) (Figure 6A), respectively. To confirm whether *bra-miR156HG* and *bra-miR5718HG* could generate corresponding miRNAs or not, the fragments of the two lncRNAs were inserted into the pBI121 vector (Figure 6B), respectively, and a transient transformation assay in tobacco was performed. Semi-quantitative RT-PCR showed that both lncRNAs were successfully expressed in tobacco leaves (Figure 6C). The expression levels of bra-miR156 and bra-miR5718 were significantly increased in leaves injected with relevant specific fragments of host genes (Figure 6D). These results proved that *bra-miR156HG* and *bra-miR5718HG* can generate mature miRNAs *in vivo*.

### Overexpression of *bra-miR5718HG* in *Brassica campestris* Upregulated *bra-miR5718* and Affected Pollen Tube Growth and Seed Set

**FIGURE 7:**
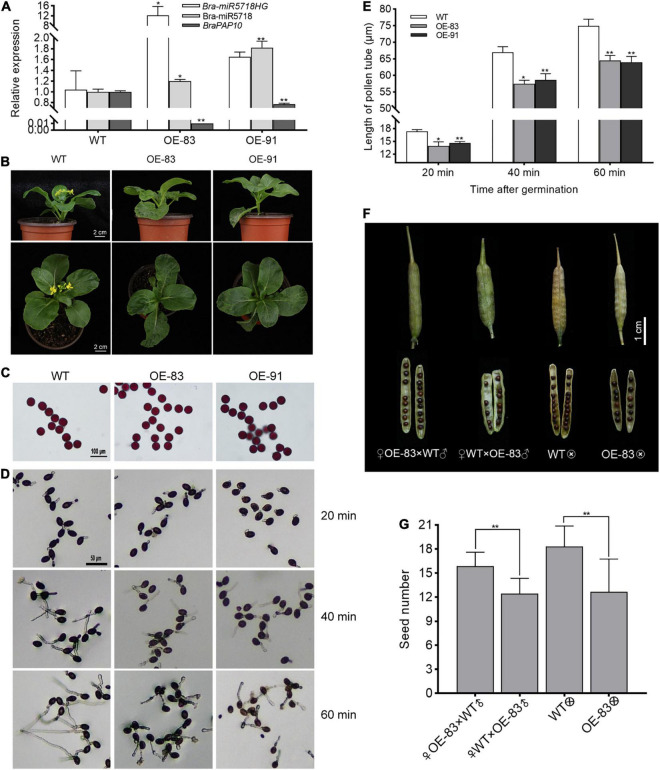
*Bra-miR5718HG* slow down pollen tube growth *via* working as host genes for miR5718 in *B. campestris*. **(A)** Relative expression of *bra-miR5718HG*, bra-miR5718, and *braPAP10* in transgenic and wild-type (WT) plants. **(B)** Photograph of *bra-miR5718HG* overexpression transgenic lines and WT. **(C)** Alexander staining of pollen grains from *bra-miR5718HG* overexpressing plants in *B. camperstris*. **(D)** Representative photograph of pollen germination *in vitro* of overexpression lines and WT. **(E)** Pollen tubes length of overexpression lines and WT at different times after germination. **(F)** Representative photograph of siliques from reciprocal cross and self-cross of transgenic and WT plants. **(G)** statistics of seed number of siliques from reciprocal cross and self-cross of transgenic and WT plants. Data were presented as mean ± SD and the *t*-test was used for significance analysis. **P < 0.01. Triplicate experiments were conducted.

To answer whether the host gene of miRNA would function in pollen development or not, we created the *bra-miR5718HG*-overexpressed transgenic plants in *B. campestris*. Two transgenic lines, namely OE-83 and OE-91, with successful overexpression were obtained. The expression of bra-miR5718 was correspondingly increased while its target gene *braPAP10* was downregulated in these lines (Figure 7A). Compared with wild type (WT), no obvious abnormality was observed during the vegetative phase, but the flowering time of transgenic plants was slightly delayed for 3 days (Figure 7B). Alexander staining of pollen grains showed that pollen viability was not affected in the two transgenic lines (Figure 7C). However, *in vitro* germination assay showed that the length of pollen tubes in transgenic plants was significantly shorter than that in WT plants, thus, a time-series *in vitro* germination assay was performed to observe the growth rate of pollen tubes in the transgenic plants. At 20 min after germination, the average pollen tube length of WT was 17.34 μm, yet it was 14.27 μm of OE-83 and OE-91 (Figures 7D,E). With the increase of germination time, the difference in pollen tube length was more obvious. The average pollen tube length of WT reached 74.95 μm at 60 min after germination, while it was only 64.27 μm of OE-83 and OE-91, which was significantly shortened (*P* < 0.01) (Figures 7D,E). Further, to see if the shorter pollen tubes affect seed set, take the line OE-83 as an example, we crossed OE-83 (♀) with WT (♂) through fertilizing the stigma of emasculated OE-83 (♀) flower buds with WT (♂) pollen grains. The resultant pollinated OE-83 (♀) flowers were 100% fertile and produced normal silique in which vigor seeds developed. By contrast, the reciprocal WT (♀) × OE-83 (♂) cross caused shorter silique and fewer seeds inside. Such infertility was also observed in OE-83 self-crossed plant but not in WT self-crossed plant. This result suggested that over-expression of *bra-miR5718HG* influenced the seed set in *B. campestris* (Figures 7F,G).

## Discussion

### Whole Transcriptome Analysis Helps Us to Deeply Understand the Gene Regulation During Anther and Pollen Development in *Brassica* Crops

Sporocyte formation and meiosis are the early stages of pollen development. *SPL/NZZ* is required for the initiation of sporogenesis in *Arabidopsis* (Wijeratne et al., 2007). In the present study, *Bra026359* (*SPL/NZZ*) were degraded in P line. Analysis of P line-specific DEGs identified candidate genes for anther cell differentiation (Figure 2A and Table 1). Most of P line-specific DEGs were transcription factors and their homologous genes in *Arabidopsis* formed a network, in which PI seemed to target most genes. PI could form a higher-order heterotetrametric complex with Agamous-like 13 (AGL13)-AG-Apetala 3 (AP3) to regulate the expression of *SPL/NZZ* (Hsu et al., 2014). Thirteen DEGs displayed the same expression changes between P line and *spl/nzz* mutant and half of them belong to the MYB or Receptor-like protein kinases (RLKs) families. MYB7, MYB56, and MYB105 were MYB family members who worked in different parts of the plant reproductive process. *AtMYB7* and its homologs *AtMYB4* affected pollen exine formation, *AtMYB56* negatively regulated the flowering time, and the double mutants of *AtMYB105* and *AtMYB117* presented disordered floral organ boundary specification, and meristem initiation and maintenance (Lee et al., 2009; Chen et al., 2015; Wang et al., 2020). Serval RLKs have been studied in anther development including *Arabidopsis Excess microsporocytes 1 (EMS1)* and *Barely any meristem 1*/*2* (*BAM1*/*2*) (Cai and Zhang, 2018). Here, three members of RLKs, *STRUBBELIG-RECEPTOR FAMILY* (*SRF2*), *Gassho2* (*GSO2*), and *C-terminally encoded peptide (CEP) receptor 2* (*CEPR2*) have also been identified (Table 1). It was more diverse for the function of the three members. *AtSRF2* negatively affected plant fertility while *GSO2* and *CEPR2* regulate the morphological architecture of plant roots through sucrose response (Eyuboglu et al., 2007; Racolta et al., 2014; Dimitrov and Tax, 2018). Although its roles in anther cell differentiation remain to be explored, *GSO2* positively regulates root cell proliferation and differentiation (Racolta et al., 2014). Interactions between these transcription factors and *SPL*/*NZZ* will create new windows of pollen development.

Whole transcriptome analysis showed that expression changes of well-known networks in anther/pollen development varied in different male sterile lines with different abortion phenotypes (Figures 4, 5). Pollen wall and tapetum are essential structures for functional pollen grains and the synthesis or regulatory pathways for them have been well studied (Parish and Li, 2010; Shi et al., 2015). In the A line and O line, most of the genes related to pectin, cellulose, and callose synthesis were downgraded. *Bra006395* (*UGP2*) was downregulated only in the two male sterile lines, A line and O line. UDP-glucose pyrophosphorylase (UGPase) catalyzes glucose-1-phosphate to UDP-glucose, which makes it a key enzyme in carbohydrate metabolism. Suppressing of *OsUGP1* and *OsUGP2* resulted in reduced callose production and delayed tapetum and middle layer degeneration, and further produced degenerated microspores (Chen et al., 2007). Strikingly, all orthologs of sporopollenin synthesis genes were downregulated in the P line. Considering that few pollen sacs were formed in the anthers of the P line as the standstill of cell differentiates, down-regulation of these genes may be caused by a decreased amount of pollen grains (An et al., 2014). The proper degradation of tapetum is essential for pollen wall formation and microspores maturation. In this study, the majority of tapetum development-related genes showed declined expression in the P line, while only genes that functioned in tapetum degradation were downregulated in the A line and O line.

### Interplays Between mRNAs and ncRNAs Are Universal During Anther and Pollen Development

Although transcriptome analysis for different male-sterile lines has been performed in Cruciferae crops, such as cabbage (Kang et al., 2008), broccoli (Shu et al., 2018), and turnip (Lin et al., 2019), the participation of ncRNAs, especially newly defined lncRNAs, and the cross-talk between mRNAs and ncRNAs still need to be explored. The whole transcriptome sequencing in this study revealed that among the transcripts identified in the three male sterile lines of *B. campestris*, mRNAs were the most abundant transcripts. However, a considerable number of lncRNAs and miRNAs were also identified. Interestingly, the proportion of downregulated mRNAs and ncRNAs was significantly higher than that of the upregulated ones (Figure 1). It was inferred that most of the expressed genes and ncRNAs play a positive role in pollen development and lower expression of them contributed to the infertility of *B. campestris*.

LncRNA-mRNA interaction was found in pollen wall construction, tapetum development, anther cell differentiation, and pollen meiosis (Figures 2–5 and Supplementary Data 2–5). Except for coding genes, lncRNA and miRNA have been proved to play a part in male sterility *via* forming complex interaction networks (Han et al., 2021). Interplays between lncRNAs and miRNAs are various. In this study, 35 DELs were predicted to be miRNA targets, eTMs, and precursors (Table 3). Among them, *bra-miR156HG* and *bra-miR5718HG* were experimentally validated to be miRNA precursors. In *Arabidopsis*, miR156 is the main factor for the phase change and is responsible for leaf morphology, trichome distribution, and lateral organogenesis (Zheng et al., 2019). Bra-miR5718 is a novel potential heat-responsive miRNA, which may also contribute to pollen development considering its relatively high expression level in *Ogu* CMS Chinese cabbages (Yu et al., 2012; Wei et al., 2015). Given the extremely significant positive correlation between the expression of two host genes and corresponding miRNAs, and the results of transient transformation assay, it can be assumed that *bra-miR156HG* and *bra-miR5718HG* generated the corresponding miRNAs *in vivo* (Figure 6).

### *Bra-miR5718HG* Negatively Regulated Pollen Tube Growth in *Brassica campestris*

In the top30 GO enrichment items, “nucleus” and “plasma membrane” were included in most of the DEGs. Furtherer, we found that 461 DEGs were enriched to the term “pollen tube growth” (Supplementary Figure 4). Our transgenic assay revealed that overexpression of *bra-miR5718HG* led to the upregulation of bra-miR5718 and reduced expression of *braPAP10* (Figure 7A). Delayed pollen tube growth *in vitro* of the transgenic plants overexpressing *bra-miR5718HG* was observed, which resulted in shorter pollen tubes and finally caused less seed set in *B. campestris* (Figures 7D–G). *AtPAP10* (*AT2G16430*), the *Arabidopsis* homolog of *braPAP10*, is a member of the purple acid phosphorylase (PAP) family (Xie and Shang, 2018). PAPs are usually involved in the acquisition and distribution of phosphorus in plants, yet more functions of them have been revealed besides Pi deficiency adaption (Bhadouria and Giri, 2021). Overexpression of *AtPAP2* improved sucrose phosphate synthase (SPS) activity and the levels of sugars and tricarboxylic acid (TCA) metabolites, resulting in earlier bolting and higher seed yield (Sun et al., 2012). Interestingly, many PAPs showed relatively higher expression in reproductive tissues, and 21 PAP genes in *Arabidopsis* were detected expression in dry pollen and germinated pollen tubes in microarray analysis (Kuang et al., 2009; Qin et al., 2009; Xie and Shang, 2018). *AtPAP10*, *AtPAP18*, and *AtPAP27* were upregulated two or threefolds in the transcriptome of pollen tubes compared to the mature pollen, which meant that they might have a positive role in pollen tube growth (Yu et al., 2012). *AtPAP15* (*At3g07130*), a PAP with phytase activity, showed an obvious GUS signal in mature pollen grains and germinated pollen tubes (Kuang et al., 2009). Consistent with its expression, mutants of *AtPAP15* presented lower pollen germination (30–35%) compared with WT (78%), which can be explained by its phytase activity since the major storage form of phosphorus in pollen grains is phytate (Kuang et al., 2009). Biochemical properties analysis of *AtPAP10* revealed that it has hydrolysis activity on ATP, ADP, dATP, pyrophosphate, polyphosphate, and phytate (Wang et al., 2011). In this study, shorter pollen tubes were observed in *bra-miR5718HG* overexpressing plants and the expression of *braPAP10* was downregulated. These results indicated that *braPAP10* may play a positive role in pollen tube growth of *B. campestris* and its expression was regulated by *bra-miR5718HG*.

Above all, it is apparent that interactions between mRNAs and ncRNAs, as well as different ncRNAs, are universal and widespread during pollen and anther development, and the discovery of these interactions can help to better understand the molecular mechanism underlying this process. Moreover, our study provides many candidate genes and ncRNAs, and further confirmation for the function and the interaction of them will unveil the complex network for pollen development.

## Data Availability Statement

The datasets presented in this study can be found in online repositories. The names of the repository/repositories and accession number(s) can be found below: https://www.ncbi.nlm.nih.gov/, PRJNA753197.

## Author Contributions

LH designed the research. DZ, ZJ, JWC, and DL analyzed the transcriptome data. DZ, CC, and TL generated the transgenic plants and performed the experiments. JSC constructed the male sterile lines. DZ, XX, SL, and LH wrote the manuscript. All authors have read and approved the manuscript.

## Conflict of Interest

The authors declare that the research was conducted in the absence of any commercial or financial relationships that could be construed as a potential conflict of interest.

## Publisher’s Note

All claims expressed in this article are solely those of the authors and do not necessarily represent those of their affiliated organizations, or those of the publisher, the editors and the reviewers. Any product that may be evaluated in this article, or claim that may be made by its manufacturer, is not guaranteed or endorsed by the publisher.

## Footnotes

1
http://www.biomarker.com.cn

2
http://brassicadb.cn/#/

3
http://www.unafold.org/RNA_form.php

4
http://www.mirbase.org/

5
http://plantgrn.noble.org/psRNATarget/
