# Supplementary material for: Transcript Profiling Analysis and ncRNAs’ Identification of Male-Sterile Systems of Brassica campestris Reveal New Insights Into the Mechanism Underlying Anther and Pollen Development
Source: Front Plant Sci. 2022 Feb 8;13:806865. doi: 10.3389/fpls.2022.806865 (PMC8861278; doi:10.3389/fpls.2022.806865)
Supplement: Supplementary file 7 [file Data_Sheet_1.docx]

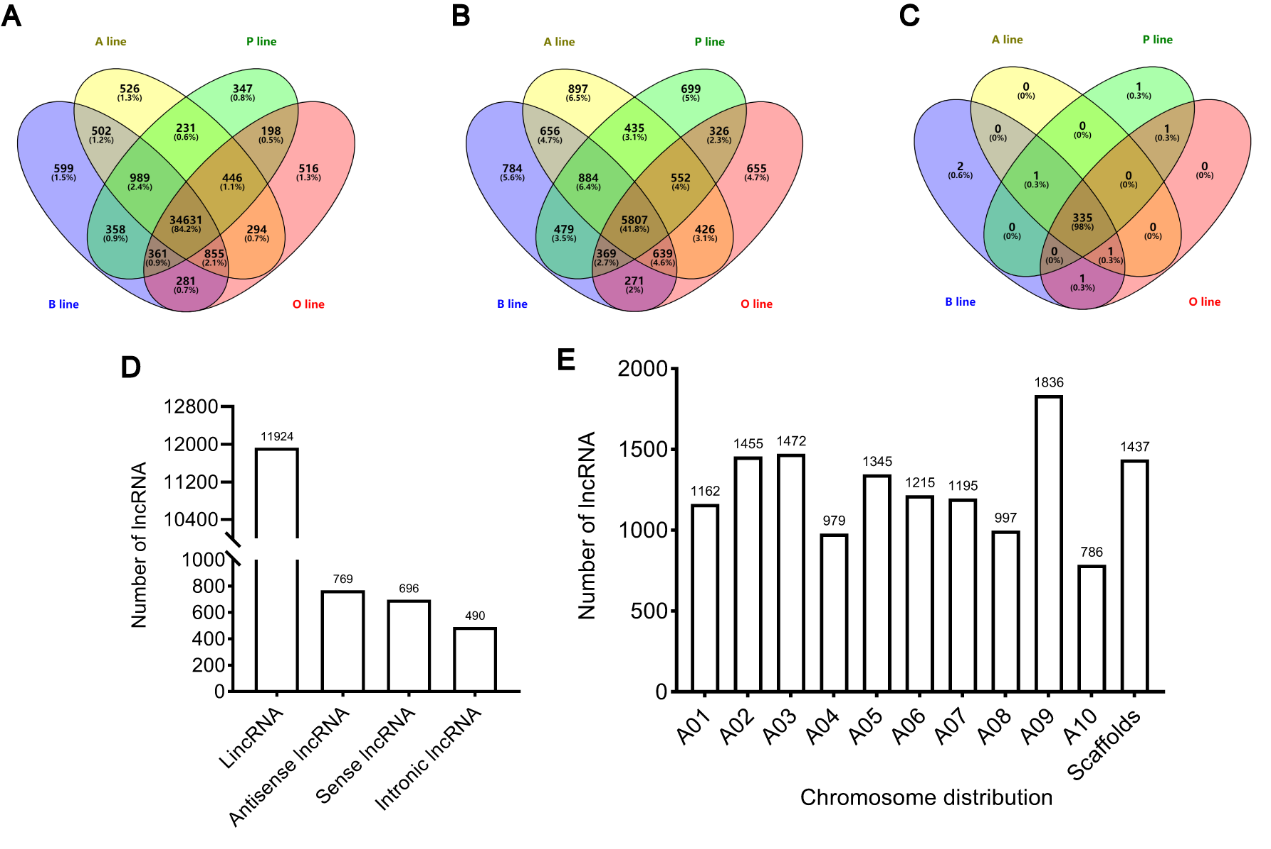


**Supplementary Figure 1 Genome-wide identification of different transcripts in *Brassica campestris*. (A)** Statistics of expressed mRNAs in three sterile lines and fertile line; **(B)** Statistics of expressed long non-coding RNAs in three sterile lines and fertile line; **(C)** Statistics of expressed microRNAs in three sterile lines and fertile line; **(D)** Statistics of lncRNAs in different types. **(E)** Chromosome distribution of identified lncRNAs


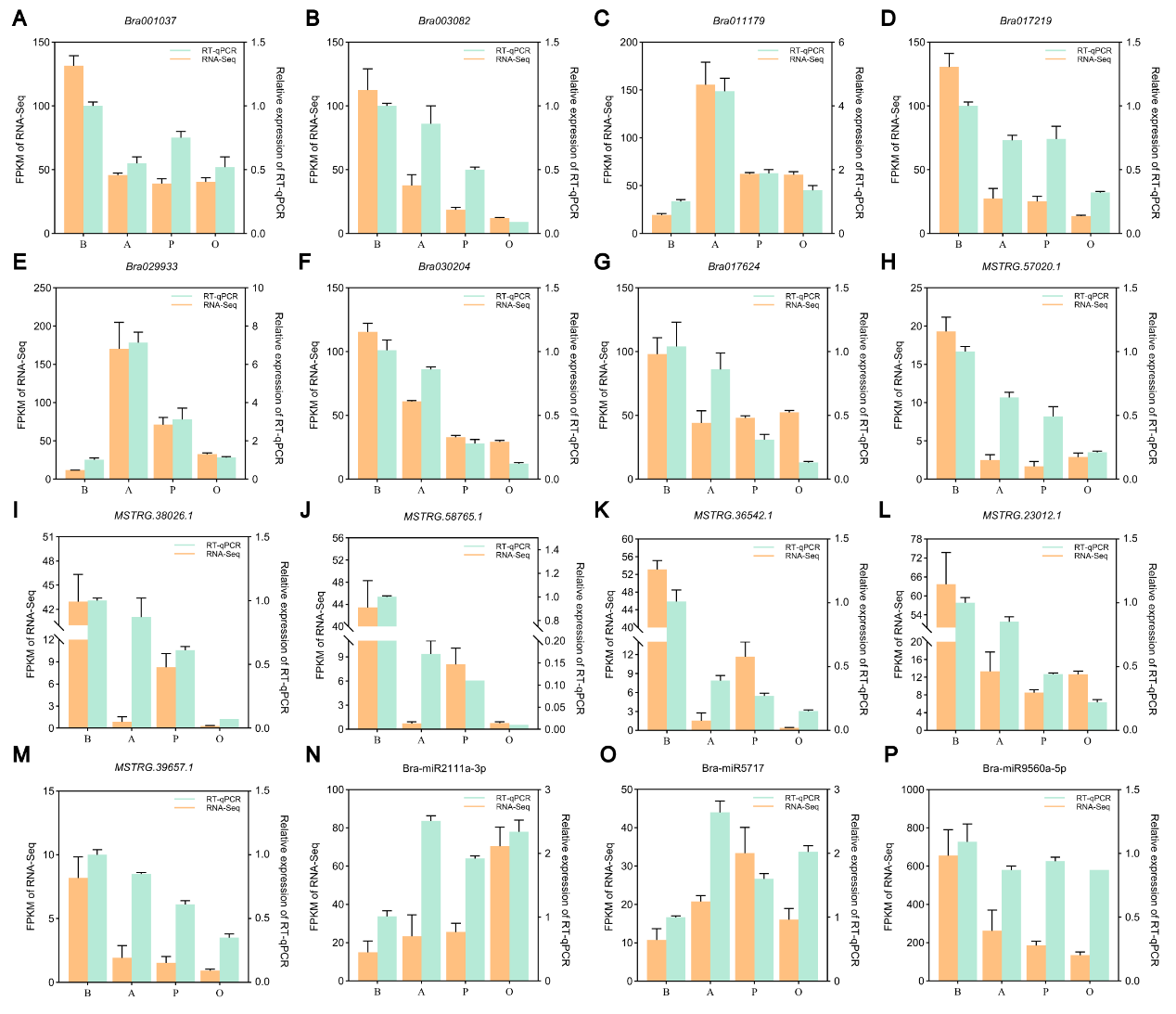


**Supplementary Figure 2 Real-time quantitive PCR (RT-qPCR) validation of selected genes from RNA sequencing.** **(A–G)** RT-qPCR results of selected differentially expressed genes. **(H–M)** RT-qPCR results of selected differentially expressed lncRNAs. **(N–P)** RT-qPCR results of selected differentially expressed miRNAs.


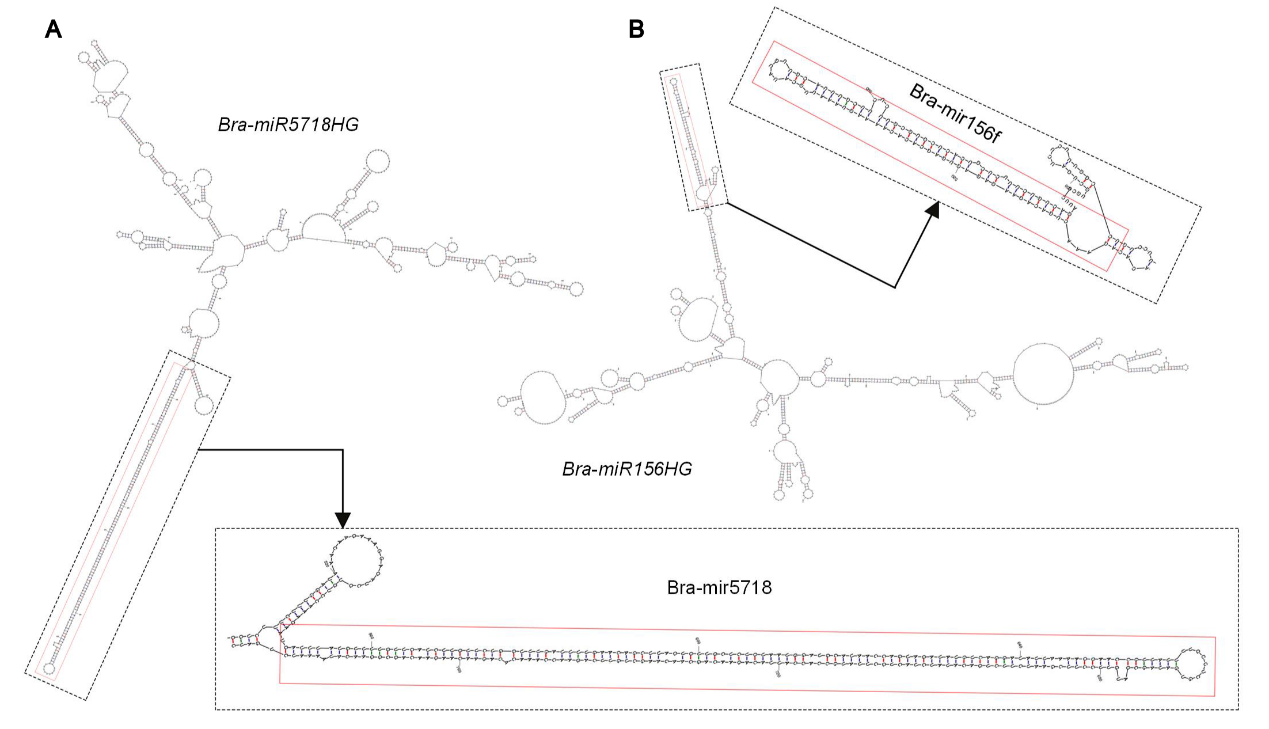


**Supplementary Figure 3 Secondary structure of two host genes in *Brassica camperstris***. **(A)** The secondary structure of bra-miR156HG, the host gene of bra-miR156. **(B)** The secondary structure of bra-miR5718HG, the host gene of bra-miR5718. The sequences in the red box indicate the predicted precursor sequence of the corresponding microRNA


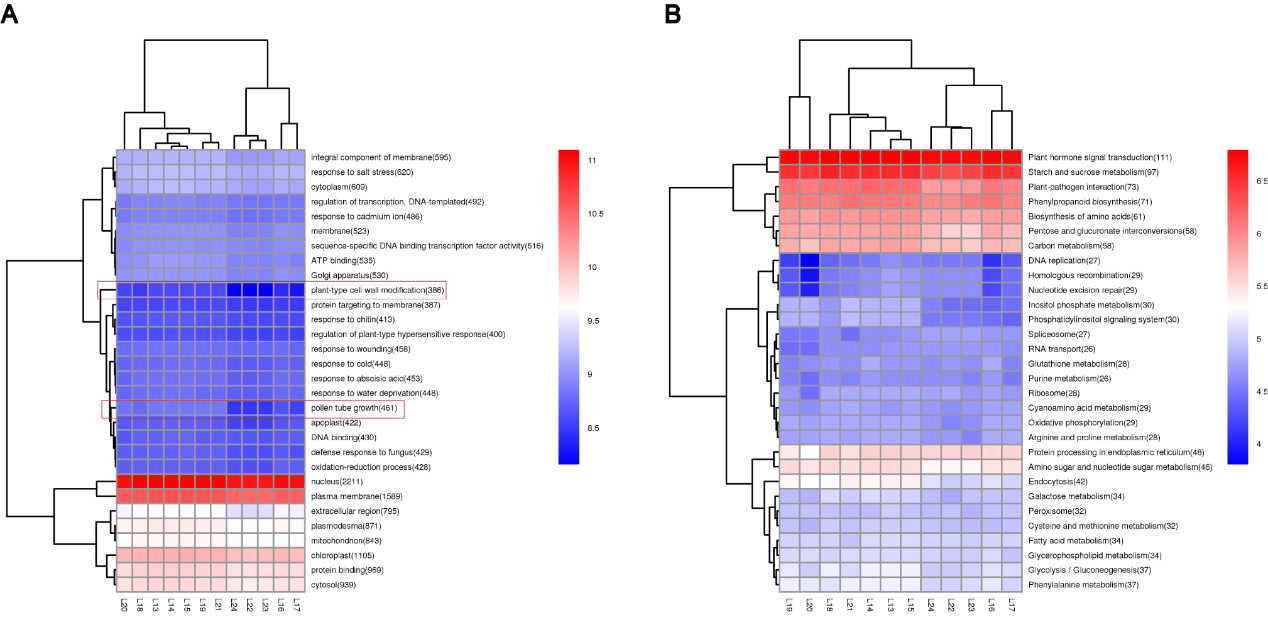


**Supplementary Figure 4 GO and KEGG enrichment analysis of inflorescences’ transcriptome in *Brassica campestris*.** **(A)** GO enrichment analysis; **(B)** KEGG enrichment analysis

**Supplementary Table 1 Primers used for RT-PCR, RT-qPCR, and transgenic plants conformation**

| Primer name | Primer sequence |
| --- | --- |
| Bra001037-qPCR-F1 | ATCCCACTGAACCACGCCG |
| Bra001037-qPCR-R1 | TTCGGACCGAGGATACGGTT |
| Bra003082-qPCR-F1 | AGCGTCCCAACATATCCAAG |
| Bra003082-qPCR-R1 | TCATGGCACCGTCTAGTTTC |
| Bra011179-qPCR-F1 | CCACACGTACTCAATCCTCTG |
| Bra011179-qPCR-R1 | CTGCGTTCCATAAACTTGCG |
| Bra017219-qPCR-F1 | AAATCGTCAGAGCCACCTG |
| Bra017219-qPCR-R1 | AAATGACCGTGAACTCCCTG |
| Bra029933-qPCR-F1 | TCCAGAGCGACCAAGAATTG |
| Bra029933-qPCR-R1 | GAGTTCCCGTAAGAGGTGTAATG |
| Bra030204-qPCR-F1 | AGATGAATCCGTTGGTGTCG |
| Bra030204-qPCR-R1 | TTCCTCTAATTGCGACTGACC |
| Bra017624-qPCR-F1 | AGACTTCTCCTACCAACGGA |
| Bra017624-qPCR-R1 | CCATCGTGTTCTTGGCGTTA |
| MSTRG.57020.1-qPCR-F1 | GGAAACTCAATGCTTGGAAAGG |
| MSTRG.57020.1-qPCR-R1 | AGTAGAGATGAAAGGCAGTGATG |
| MSTRG.38026.1-qPCR-F1 | TCTCGTCCGTATTGTTCTTGTG |
| MSTRG.38026.1-qPCR-R1 | TGTAGCTCGACATGGAAAAGG |
| MSTRG.58765.1-qPCR-F1 | TGATCCAGAGCGGTTCGTTC |
| MSTRG.58765.1-qPCR-R1 | TCGCAAGAAGCTCGTCAAGT |
| MSTRG.36542.1-qPCR-F1 | AGTCCAAAACTGCTAAAAAGAA |
| MSTRG.36542.1-qPCR-R1 | GAAGCTCCTCATTGTAAGAAC |
| MSTRG.23012.1-qPCR-F1 | CAGGTTAGGAGTTGAGGCATG |
| MSTRG.23012.1-qPCR-R1 | CCAACCTCACGCAGTCTC |
| MSTRG.39657.1-qPCR-F1 | GGAAACAAATTACGCAGCCG |
| MSTRG.39657.1-qPCR-R1 | GTCGCACACTTGGTCATTAAG |
| Bra-miR2111a-3p | GTCCTCGGGATGCGGATTACC |
| Bra-miR5717 | GTTTGGATTGTTTGCCTTGGC |
| Bra-miR9560a-5p | ACAGGTGGTGGAACAAATATGAGT |
| Bra-miR156HG2-121-HR-F2 | catttacgaacgatatctagaTGCCTCGGCAGACATCTGTT |
| Bra-miR156HG2-121-HR-R2 | ataagggactgaccacccgggTCGAGAAATTAAGGACCAGCCT |
| Bra-miR5718HG-121-HR-F2 | catttacgaacgatatctagaCCGTTCTGGTTTGGAGAGTGT |
| Bra-miR5718HG-121-HR-R2 | ataagggactgaccacccgggCCGTTCTGGTTTGGAGAGTGT |
| 156hg2-qPCR-F | TCTTGTAGATCTCTAGAGTTGGATG |
| 156hg2-qPCR-R | ACAAGCCATCATTTCAGTGGT |
| 5718hg-qPCR-F | CAAGAAAGGAGATCGTGTCCAA |
| 5718hg-qPCR-R | AATTTCCCAAGGGTCGTGTGT |
| 18sRNA-F2 | AGGATTGACAGACTGAGAGC |
| 18sRNA-R2 | CACAGACCTGTTATTGCCTC |
| Bra-miR156c-5p | TGACAGAAGAGAGTGAGCAC |
| Bra-miR5718c-5p | TCAGAACCAAACACAGAACAAG |
| braPAP10-1-qF1 | GCGTCTCGAAGGACCAAATC |
| braPAP10-1-qR1 | AACCGTTGCCTCTAACCTCC |
| 5.8S-F | CTCTCGGCAACGGATAT |
| 5.8S-R | CTGCAATTCACACCAAGT |
| pBI121-35s-GUS-F2 | GATTGATGTGATATCTCCACTGAC |
| pBI121-35s-GUS-R2 | TTCTACAGGACGTAACATAAGG |
